# The relationship between headache and religious attendance (the Nord-Trøndelag health study- HUNT)

**DOI:** 10.1186/1129-2377-15-1

**Published:** 2014-01-03

**Authors:** Erling Tronvik, Torgeir Sørensen, Mattias Linde, Lars Bendtsen, Ville Artto, Katarina Laurell, Mikko Kallela, John-Anker Zwart, Knut Hagen

**Affiliations:** 1 Norwegian National Headache Centre, Trondheim University Hospital, Trondheim, Norway; 2 Department of Neurosciences, Norwegian University of Science and Technology, Trondheim, Norway; 3 MF Norwegian School of Theology, Oslo, Norway; 4 Department of Neurology, Danish Headache Centre, Glostrup Hospital, Glostrup, Denmark; 5 University of Copenhagen, Copenhagen, Denmark; 6 Department of Neurology, Helsinki University Hospital, Helsinki, Finland; 7 Department of Pharmacology and Clinical Neuroscience, Östersund Hospital, Östersund, Sweden; 8 Umeå University, Umeå, Sweden; 9 Department of Neurology and FORMI, Oslo University Hospital and University of Oslo, Oslo, Norway; 10 Department of Neurology, Trondheim University Hospital, Trondheim, Norway

**Keywords:** Epidemiology, Religion, Spirituality, Headache, Migraine

## Abstract

**Background:**

Religious belief can be used as a pain coping strategy. Our purpose was to evaluate the relationship between headache and religious activity using prospective data from a large population-based study.

**Methods:**

This longitudinal cohort study used data from two consecutive surveys in the Nord-Trøndelag Health Survey (HUNT 2 and 3) performed in 1995–1997; and 2006–2008. Among the 51,383 participants aged ≥ 20 years who answered headache questions at baseline, 41,766 were eligible approximately 11 years later. Of these, 25,177 (60%) completed the question in HUNT 3 regarding religious activity. Frequent religious attendees (fRA) (used as a marker of stronger religious belief than average) were defined as those who had been to church/prayer house at least once monthly during the last six months.

**Results:**

In the multivariate analyses, adjusting for known potential confounders, individuals with headache 1–14 days/month in HUNT 2 were more likely to be fRA 11 years later than headache-free individuals. Migraine at baseline predisposed more strongly to fRA at follow-up (OR = 1.25; 95% CI 1.19-1.40) than did non-migrainous headache (OR = 1.13; 95% 1.04-1.23). The odds of being fRA was 48% increased (OR 1.48; 95% 1.19-1.83) among those with migraine 7–14 days/month at baseline compared to subjects without headache. In contrast, headache status at baseline did not influence the odds of being frequent visitors of concerts, cinema and/or theatre at follow-up 11 years later.

**Conclusions:**

In this prospective study, headache, in particular migraine, at baseline slightly increased the odds of being fRA 11 years later.

## Background

A positive relationship between religiousness/spirituality and mental and physical health measures has been demonstrated mainly in cross-sectional studies [1]. Religious activity may be one of several pain coping strategies to manage suffering. This is illustrated by one study reporting that 40% of patients with different types of chronic pain reported that religion and spirituality had become more important in their lives, and that prayer had a beneficial effect on pain tolerance [2].

Headache can severely affect the quality of life and the socioeconomic costs to society [3]. To our knowledge, no previous studies have evaluated the relationship between headache and religious activity using a prospective study design.

The aim of the study was to evaluate whether headache at baseline influenced the occurrence of frequent religious activity at follow-up in a large prospective study.

## Methods

### The HUNT study

The Nord-Trøndelag Health Survey (HUNT) is a longitudinal cohort study in which all inhabitants ≥ 20 years old in Nord-Trøndelag were invited to participate. Subjects were examined three times; from 1984 to 1986 (HUNT 1), 1995 to 1997 (HUNT 2) and 2006 to 2008 (HUNT3). The surveys covered a large number of health-related items, and participants were also invited to clinical consultations, which included blood samples and measurements of blood pressure, height and weight. HUNT 2 included questions on headache, whereas HUNT 3 also included question on religious attendance.

### Sampling frame

Nord-Trøndelag is one of 19 counties in Norway, and is fairly representative for the rest of the country. During HUNT 3, 89.7% of the inhabitants were members of the evangelical Lutheran Church, 2.1% were members of other Christian churches, and 0.4% belonged to other religions [4]. Norwegians are registered at baptism as members of the Church of Norway.

### HUNT 2 questionnaire

In HUNT 2 each person completed extensive questionnaires eliciting information on health problems. Among a wide range of topics in the first questionnaire (Q1) were education, physical activity, smoking, and anxiety and depression (measured by the Hospital Anxiety and Depression Scale (HADS)). Details of the phrasing of these questions have been described previously [5-8]. Educational level was categorized according to duration: ≤9 years, 10–12 years, and ≥13 years. Cigarette smoking was categorized as “current daily smoking”, “previous daily smoking”, and “never daily smoking”. Reponses to questions on physical activity were categorized according to duration and intensity of exercise per week: ≥3 hours hard physical activity, 1–2 hours hard physical activity, ≥3 hours light physical activity, 1–2 hours light physical activity and physical inactivity (0 hours). BMI was subdivided into three groups: <25 kg/m^2^, 25–29.9 kg/m^2^ and ≥30 kg/m^2^.

The headache questions in the second questionnaire (Q2) were designed principally to determine whether or not each person had headache and, if so, its frequency, and to diagnose migraine according to a modified version of the first version of the International Classification of Headache Disorders (ICHD-I) [9] when headache was reported. Subjects who answered “yes” to the screening question “Have you suffered from headache during the last 12 months?” were classified as having headache. Mutually exclusive diagnoses were made for migraine and non-migrainous headache [10]. Chronic daily headache (CDH) was defined as headache occurring on ≥ 15 days/month.

The validity of these questionnaire-based diagnoses was reported previously [6,10]: for headache sensitivity was 85% and specificity 83% (kappa value 0.57); for migraine, sensitivity was 69% and specificity 89% (kappa value 0.59); for non-migraineurs, sensitivity was 61%, specificity 81% (kappa 0.43) and for CDH, sensitivity was 38%, specificity 97% (kappa 0.44).

### HUNT 3 questionnaire

HUNT 3 was to a large extent a replication of HUNT 2 but included also a question on religious attendance (RA) in Q2 measured with the item “How often in the last 6 months have you been to church/prayer house?” with possible responses covering “never”, 1–6 times in the last 6 months”, “1-3 times/month”, and “more than 3 times/month”. In the prospective analysis, frequent religious attendees (fRA) were defined as those who answered “1-3 times/month” or “more than 3 times/month”. The individuals were also asked to respond to frequency of non-religious social activities using the question “How often in the last 6 months have you been to concerts, cinema and/or theatre” with similar response options. Frequent social activity attendees (fSA) were defined as those who answered “1-3 times/month” or “more than 3 times/month”.

The Q2 also included the same headache screening question that was used in HUNT 2. For the questionnaire-based status as a headache sufferer, a sensitivity of 88%, a specificity of 86%, and a kappa value of 0.70 were found [11].

### Samples and procedures

**Figure 1:**
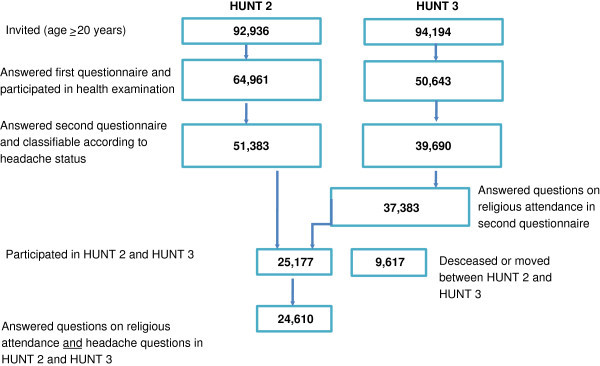
Flow diagram of the study population.

In HUNT 2, 51,383 (56%) of 92,936 invited individuals answered the headache-related questions in Q2. Details of non-responders have been described previously [10]. In HUNT 3, 50,839 (54%) of the 94,194 invited adults answered Q1 and 37,383 (40%) answered the question in Q2 on RA. Details of non-responders have been described previously [11]. The mean time of follow-up was 11.3 (range 9–13) years. Among the 51,383 who answered the headache questions in HUNT 2, 6,608 had died and 3,009 had moved out of the county by the time of HUNT 3, leaving 41,766 eligible individuals for this analysis. Of these, 25,177 (60%) completed the questions regarding RA in HUNT 3 whereof 24,610 answered headache questions both in HUNT 2 and HUNT 3. The flow chart of participants in HUNT 2 and HUNT 3 is shown in Figure 1.

### Statistical analysis

By multivariate analyses using logistic regression we estimated odds ratio (OR) with 95% confidence intervals (CI) evaluating the influence of headache status at baseline in HUNT 2 on the odds of being fRA in HUNT 3. The relationship between headache in HUNT 2 and the four RA categories was also evaluated, and these categories were treated as a single ordinal variable and incorporated in a two-sided test for trend to evaluate the probability of a linear relationship between RA categories and headache. The trend test was considered statistically significant at p < 0.05. We also evaluated potential confounding factors identified previously [5,6,8,12-14]: in particular age (continuous variable), gender, education level, smoking status, physical activity, body mass index, chronic musculoskeletal complaints, systolic blood pressure (SBP) and anxiety and depression measured by total HADS score (continuous variable). Subjects with incomplete data for one or several variables were included (as a separate missing category) in all analyses to reduce the impact of response bias. In supplementary analyses, we evaluated the influence of headache status in HUNT 2 and HUNT 3 on the odds of being fRA in HUNT 3. Statistical analyses were performed with the Predictive Analytics SoftWare (PASW) Statistics version 17.0 by SPSS inc., an IBM Company (Chicago, IL).

### Ethics

The Norwegian Data Inspectorate, the Norwegian Board of Health, and the Regional Committee for ethics in Medical Research had approved all HUNT studies, and the Regional Committee also approved the present analysis.

## Results

Demographic and clinical characteristics at baseline in HUNT 2 are given in Table 1 separated by frequency of RA in HUNT 3. Individuals who visited church/prayer house 1–3 times/month or more than 3 times/month were older than those with less frequent visits. For both sexes differences between groups were found regarding e.g. education, depression as measured by HADS, BMI and SBP. Adjustments for all these potential confounding factors were made in the multivariate analyses.

**Table 1:** Baseline data for the different church attendance frequencies

**Variables**	**Never**	**1-6 times/last 6 months**	**1-3 times / month**	**More than 3 times/month**	**p**
**Men**					
n	4171	5249	1083	377	
Mean age, year (SD)	46.4 (12.8)	47.0 (12.4)	54.4 (12.4)	52.4 (12.4)	P < 0.001
HADS-A^a^ (SD) (missing^f^ = 770)	3.8 (3.1)	3.8 (2.9)	3.8 (3.0)	4.0 (3.0)	P = 0.612
HADS-D^b^ (SD) (missing^f^ = 430)	3.5 (3.0)	3.4 (2.8)	3.7 (2.9)	3.7 (2.9)	P = 0.001
HADS-total^c^ (SD)	7.2 (5.3)	7.2 (4.9)	7.3 (5.1)	7.6 (5.2)	P = 0.351
SBP^d^ mean (SD) (missing^f^ = 40)	137.5 (16.6)	136.9 (16.0)	140.7 (18.2)	138.3 (17.9)	P < 0.001
Education > 12 years (%)	24.3	24.6	18.5	24.9	P < 0.001
Physical inactivity (%)	5.2	3.8	3.9	5.8	P < 0.001
BMI^e^, mean (SD) (missing^f^ = 13)	26.4 (3.4)	26.4 (3.1)	26.7 (3.2)	26.5 (3.2)	P = 0.014
Smoking (%)	27.3	21.4	17.6	16.2	P < 0.001
**Women**					
n	5141	6930	1672	554	
Mean age, year (SD)	44.4 (13.1)	45.0 (13.0)	53.5 (13.1)	52.6 (13.2)	P < 0.001
HADS-A^a^ (SD) (missing^f^ = 1592)	4.4 (3.4)	4.3 (3.2)	4.4 (3.3)	4.3 (3.4)	P = 0.46
HADS-D^b^ (SD) (missing^f^ = 707)	3.1 (3.0)	3.0 (2.8)	3.5 (2.9)	3.2 (2.8)	P < 0.001
HADS-tot^c^ (SD)	7.4 (5.7)	7.2 (5.3)	7.8 (5.5)	7.4 (5.5)	P = 0.005
SBP^d^ mean (SD) (missing^f^ = 24)	130 .1 (19.7)	129.8 (18.9)	137.5 (21.7)	135.7 (22.6)	P < 0.001
Education > 12 years (%)	22.9	25.5	17.7	30.7	P < 0.001
Physical inactivity (%)	4.4	2.6	3.5	6.1	P < 0.001
BMI^e^, mean (SD) (missing^f^ = 41)	25.7 (4.2)	25.8 (4.2)	26.7 (4.3)	26.9 (4.7)	P < 0.001
Smoking (%)	33.7	23.8	17.2	10.8	P < 0.001

^a^Hospital Anxiety and Depression Scale – Anxiety score ^b^Hospital Anxiety and Depression Scale – Depression score ^c^HADS total score (HADS-A + HADS-D) ^d^systolic blood pressure (mm Hg) ^e^body mass index ^f^Number of individuals with missing values are given if different from zero.

Individuals who suffered from headache at baseline in HUNT 2 were more likely to be fRA at follow-up 11 years later than headache-free (OR = 1.13, 95% CI 1.05-1.22). This relationship was more evident among men (OR = 1.18; 95% CI 1.04-1.34) than women (OR 1.11; 95% CI 1.00-1.22) (Table 2), and more evident for migraine (OR = 1.25; 95% CI 1.19-1.40) than non-migrainous headache (OR = 1.13; 95% CI 1.04-1.23).

**Table 2:** **Odds ratio (OR)**^**# **^**of frequent religious attendance (FRA) in HUNT 3 according to headache status at baseline in HUNT 2**

	**Total**	**Both genders**		**Men**		**Women**	
	**25,177**	**n**	**OR (95% CI)**	**n**	**OR (95% CI)**	**n**	**OR (95% CI)**
No headache	14,510	2166	1.00 (Ref.)	1,002	1.00 (Ref)	1,164	1.00 (Ref)
Headache	10,667	1520	1.13 (1.05-1.22)	458	1.18 (1.04-1.34)	1,062	1.11 (1.00-1.22)
< 7 days/month	8,722	1,218	1.18 (1.09-1.28)	377	1.20 (1.05-1.37)	841	1.11 (1.00-1.23)
7-14 days/month	1406	217	1.30 (1.12-1.52)	52	1.17 (0.86-1.60)	165	1.24 (1.03-1.50)
> 14 days/month	539	85	1.17 (0.92-1.47)	29	1.11 (0.73-1.68)	56	1.41 (0.85-1.52)
Migraine	3,419	491	1.25 (1.19-1.40)	120	1.22 (0.99-1.50)	371	1.19 (1.04-1.36)
< 7 days/month	2,644	366	1.23 (1.10-1.38)	98	1.26 (1.04-1.53)	268	1.16 (1.01-1.44)
7-14 days/month	609	96	1.48 (1.19-1.83)	17	1.46 (1.00-2.23)	79	1.51 (1.11-1.87)
> 14 days/month	166	29	1.57 (1.08-2.28)	5	1.06 (0.44-2.57)	24	1.64 (1.08-2.48)
Non-migrainous headache	7,248	1029	1.13 (1.04-1.23)	338	1.16 (1.01-1.33)	691	1.06 (0.95-1.19)
< 7 days/month	6078	852	1.17 (1.07-1.28)	279	1.19 (1.02-1.37)	573	1.11 (0.99-1.25)
7-14 days/month	797	121	1.15 (0.93-1.41)	35	1.14 (0.79-1.66)	86	1.09 (0.83-1.37)
> 14 days/month	373	56	0.99 (0.74-1.34)	24	1.15 (0.73-1.81)	32	0.87 (0.59-1.29)

^#^Adjusted for age, gender, educational level and chronic musculoskeletal complaints. Other potential confounders were found to have little impact on the model.

As demonstrated by Table 2, having suffered from headache less than 15 days/month at baseline, in particular headache 7–14 days/month, predisposed to being fRA 11 years later (OR = 1.30; 95% CI 1.12-1.52). This relationship was more clearly demonstrated for migraine than non-migrainous headache. Thus, the odds of fRA was 48% increased (OR 1.48; 95% 1.19-1.83) among those with migraine 7–14 days/month at baseline, more evident for women (OR = 1.51; 95% CI 1.11-1.87) than for men (OR = 1.46; 95% CI 1.00-2.23). Individuals with non-migrainous headache < 7 days/month at baseline were more likely to be fRA at follow-up (OR = 1.17; 95% CI 1.07-1.28) than headache-free, evident for men (OR = 1.19; 95% CI 1.02-1.37) but not for women (OR = 1.11; 95% CI 0.99-1.25).

In the multivariate analyses, adjusting for known potential confounders, headache status at baseline did not influence the occurrence of being fSA (Table 3).

**Table 3:** **Odds ratio (OR)**^**# **^**of frequency of visiting concerts, cinema and/or theatre (fSA) in HUNT 3 according to headache frequency at baseline in HUNT 2 (n = 25,111)**

	**Total**	**Both genders**	**Men**	**Women**
	**25,11**	**OR (95% CI)**	**OR (95% CI)**	**OR (95% CI)**
No headache	14,510	1.00 (ref.)	1.00 (ref.)	1.0 (ref.)
Headache 1–6 days/month	8,722	1.09 (0.85-1.40)	0.78 (1.05-1.19)	1.28 (0.94-1.76)
Headache 7–14 days/month	146	1.08 (0.84-1.39)	0.80 (0.87-1.23)	1.25 (0.92-1.72)
Headache ≥ 15 days/month	539	1.02 (0.76-1.36)	0.80 (0.76-1.25)	1.18 (0.83-1.68)

^#^Adjusted for age, gender, body mass index, chronic musculoskeletal complaints, smoking, educational level, systolic blood pressure, physical activity and total HADS score.

In analyses evaluating the influence of headache status in HUNT 2 and HUNT 3, headache complaints in HUNT 2 was more important than headache status in HUNT 3 on the association with fRA (both genders merged, Table 4). Among men those with headache in both surveys were more likely to be fRA (OR = 1.22; 95% CI 1.03-1.44) than headache-free in both surveys. In addition, there was a trend of increasing headache prevalence in HUNT 2 with increasing frequency of RA, evident for both genders merged and for men (Table 5).

**Table 4:** **Odds ratio (OR)**^**# **^of frequent religious attendance (fRA) according to headache status in HUNT 2 and HUNT 3 (n = 24,610^*^)

			**Frequent religious attendance (fRA)**		
**Headache**		Total number*	**Number**	**OR**	**(95% CI)**^**§**^
**HUNT 2**	**HUNT 3**				
*Overall*					
No	No	12,058	1,845	1.00	Reference
No	Yes	2,212	259	1.05	(0.91-1.21)
Yes	No	4,255	652	1.18	(1.06-1.30)
Yes	Yes	6,085	780	1.18	(1.07-1.30)
*Men*					
No	No	6,385	866	1.00	Reference
No	Yes	975	113	1.13	(0.91-1.40)
Yes	No	1,536	216	1.16	(0.98-1.37)
Yes	Yes	1,818	221	1.22	(1.03-1.44)
*Women*					
No	No	5,673	979	1.00	Reference
No	Yes	1,237	146	0.97	(0.80-1.17)
Yes	No	2,719	436	1.12	(0.98-1.27)
Yes	Yes	4,267	559	1.09	(0.96-1.23)

^#^Adjusted for age, gender, chronic musculoskeletal complaints and education. Other potential confounders were found to have little impact on the model.

^§^CI denotes confidence interval.

^*^Missing data: Incomplete headache data in HUNT-3 in 567 individuals.

**Table 5:** **The relationship between headache in HUNT 2 and church attendance frequency in HUNT 3 (n = 25,073)**^**#**^

**Church attendance frequency**	**Total**	**Both genders**	**Men**	**Women**
	**25,073**	**OR (95% CI)**	**OR (95% CI)**	**OR (95% CI)**
Never	9,276	1.00 (ref.)	1.00 (ref.)	1.0 (ref.)
1-6 times last six months	12,143	1.04 (0.98-1.10)	1.07 (0.97-1.17)	1.01 (0.94-1.09)
1-3 times/month	2727	1.10 (1.00-1.21)	1.14 (0.97-1.33)	1.08 (0.96-1.22)
More than 3 times/month	927	1.27 (1.09-1.47)	1.41 (1.12-1.78)	1.19 (0.98-1.43)
P trend		P < 0.001	0.003	0.07

^#^Adjusted for age, gender, body mass index, smoking, educational level, systolic blood pressure, physical activity and total HADS score.

## Discussion

To the best of our knowledge, this is the first population-based follow-up study evaluating the relationship between headache and RA, demonstrating that headache, in particular migraine, at baseline slightly increased the likelihood for being fRA 11 years later.

Previous studies evaluating the relationship between headache and RA are lacking. However, it may be of some relevance that a greater reduction in the number of headaches has been found in migrainours who practiced spiritual meditation compared to those practicing secular meditation or muscle relaxation exercises [15]. There are studies reporting that religious activities may be a coping strategy among patients with unspecified chronic pain [16]. In one study, 40% of patients with chronic pain reported that religion and spirituality had become more important in their lives [2]. Furthermore, previous research among older patients with chronic, non-cancer pain have listed analgesic medication (78%), exercise (35%), cognitive methods (37%), religious activities (21%) and activity restriction (20%) as the most common coping strategies [17]. In one small, uncontrolled study among patients with chronic pain secondary to sickle cell disease, church attendance was significantly associated with pain reduction [18]. Private religious activity (prayer/bible study) did, however, not show the same association. When both pain severity and tolerance were assessed, religious coping appeared to increase pain tolerance to a larger degree than the change in pain severity [19]. Interestingly, spirituality has in recent years established its role as a significant modulatory factor of pain transmission [20]. Using a cold pressor stress response test in healthy subjects, blood pressure, pulse and serum cortisol were less increased in religious subjects than in non-religious subjects when exposed to acute painful stress [21]. Further support of stress alleviation includes the association between RA and low blood pressure [22] and between spirituality and lower urinary cortisol excretion in chronic pain patients [23]. An interesting explanation of the association between migraine and RA may be the possibility that spirituality is a way to reduce the allostatic load (biological consequences of chronic exposure to repeated or chronic stress responses) that has been suspected in migraine and other pain conditions [24]. In support of this, weekly religious service attendance has been shown to lower the allostatic load among high-functioning elderly women, using different biomarkers in blood and urine samples [25].

In the present study, headache 1–14 days/month, in particular migraine, was associated with fRA at end of follow-up. A similar tendency was found for migraine >14 days/month, but the level of significance was not met. This may be due to the low number of subjects in these subgroups. An alternative explanation may be that in these subjects the potential benefit of attending RAs is overshadowed by the strain of attending these ceremonies.

A possible explanation of the relationship between headache and fRA could be that headache patients visit church regularly due to access to a social network [26]. On the other hand, no relationship between headache and non-religious social activities was found. Attending church may be a way of coping with the pain, but other mechanisms may also be involved, like predisposing personality traits, underlying genetic susceptibility or other factors. Whether church attendance is the cause of headache or provides relief cannot be definitely determined in this study, but based on the previously mentioned literature on the subject, the latter is most likely.

Stratifying by gender (Table 2), men tended to have higher fRA compared to women. This is an interesting finding as in American research literature women normally are more religious active and also benefit more than men from this activity [26]. The former is also the picture in Norway regarding frequency distribution of RA [4], but women seem to have a kind of a durative approach to religious activity, maintaining the same level of religiousness independent of demanding events and/or illness. Norwegian men, on the other hand, tend to activate religiousness when demanding events and/or illness occur [27,28].

### Strengths and limitations of the study

The major strengths of this study are the prospective design, a follow-up period of 11 years and the large cohort recruited from the adult population of an entire county. Furthermore, we had validated the questionnaire-based headache diagnoses [10,11], and evaluated the understanding of RA in the HUNT study [4]. In the multivariate analyses, we were able to adjust for a large number of potential confounding factors, but the possibility of residual confounding by an unrecognized factor cannot be ruled out. Some limitations should be considered. Firstly, information about RA at baseline was lacking. However, the typical way of defining a population at risk (never visiting church in HUNT 2) is probably not appropriate. Interestingly, we found that headache status in HUNT 2 had more impact on RA in HUNT 3 than headache status in HUNT 3. This may support the notion that RA is a coping strategy of headache complaints in HUNT 2. This may also be indicated by the fact that a dose-relationship was found between headache in HUNT 2 and frequency of RA in HUNT 3. Secondly, there was a relatively low participation rate (56%) at baseline in HUNT 2 and to the question regarding RA in HUNT 3 (60%) so that a selection bias cannot be ruled out. Despite this, the wide scope of the HUNT studies (they addressed a broad range of medical disorders) made biases with relevance to headache or RA unlikely. Thirdly, we had only one measure of religious attendance frequency. However, RA is used internationally as a measure of religiousness [29]. Fourthly, statistical power and capacity to detect significant differences, was lower for men than for women; and particularly for CDH due to the low number of subjects in these subgroups.

## Conclusion

A relationship between headache at baseline and frequently attaining religious activities was demonstrated in a large population and the association was somewhat more evident for migraine than for non-migrainous headache.

## Competing interests

ET has received fee for work as site clinical investigator in a study initiated by GSK. LB has received payment as advisory board member of Berlin-Chemie and for lectures for Allergan. MK has received payment as advisory board member of Allergan and Genzyme and paid lectures by MSD, Bayer, Orion, Boehringer-Ingelheim, Pfizer, Menarini, Leiras, GlaxoSmithKline, AstraZeneca, Janssen-Cilag, Sandoz and Meda. All authors except TS have been reimbursed by Allergan the costs of travel and hotel for 2 meetings with the co-authors of this study.

## Authors’ contributions

ET and KH performed the statistical analyses. All authors contributed in writing the paper. All authors read and approved the final manuscript.
